# Effects of sodium polyacrylate on water retention and infiltration capacity of a sandy soil

**DOI:** 10.1186/2193-1801-2-S1-S11

**Published:** 2013-12-11

**Authors:** Wenhua Zhuang, Longguo Li, Chao Liu

**Affiliations:** State Key Laboratory of Hydraulic and Mountain River Engineering, College of hydraulic and hydroelectric engineering, Sichuan University, Chengdu, Sichuan China; Institute of Soil and Water Conservation, MWR&CAS, Yangling, Shaanxi China

**Keywords:** sodium polyacrylate, water retention, infiltration, sandy soil, leakage

## Abstract

Based on the laboratory study, the effects of sodium polyacrylate (SP) was investigated at 5 rates of 0, 0.08, 0.2, 0.5, and 1%, on water retention, saturated hydraulic conductivity(Ks), infiltration characteristic and water distribution profiles of a sandy soil. The results showed that water retention and available water capacity effectively increased with increasing SP rate. The Ks and the rate of wetting front advance and infiltration under certain pond infiltration was significantly reduced by increasing SP rate, which effectively reduced water in a sandy soil leaking to a deeper layer under the plough layer. The effect of SP on water distribution was obviously to the up layer and very little to the following deeper layers. Considering both the effects on water retention and infiltration capacity, it is suggested that SP be used to the sandy soil at concentrations ranging from 0.2 to 0.5%.

## Introduction

Due to its high sand content and little clay content, which means big inter-granular space, sandy soil has very low water-holding capacity, which results in the easy deep percolation of water and the diffusion of water through macro-pore to surface and finally the evaporation; Meanwhile the capillary of sandy soil is relatively thick, which leads to the low ascending height of capillary water, so it is very difficult to depend on the ascending effect of groundwater through capillary to back moisten the surface soil. And therefore it is very important to improve the water-holding capacity of sandy soil itself and its permeability characteristics so as to enhance the water supply capacity and water use efficiency. Due to its special molecular structure and plenty of hydrophilic groups, water retaining agent, also called soil amendment, super absorbent resin and polymer, can have a retention of pure water with the weight of hundreds of and even thousands of times as water retaining agent itself and meanwhile modify soil structure through bonding soil particle and swelling by soil itself. As a sort of novel adjustment measure water retaining agent has been proposed and applied to modify soil and improve soil water-holding capacity and has been emphasized universally all over the world. Sodium polyacrylate with ultra-high molecular, which is one of the water retaining agents, has stronger water absorbent capacity, higher water absorption rate and lower price, and so has a wide application prospect.

Through a simulation test Sivapalan found out that after the application of crosslinked polyacrylamide into sandy soil by 0.03% and 0.07% respectively and at the water suction of 0.01 Mpa the moisture capacity increased by 23% and 95% respectively in comparison with control[1]. Through study Feng Hao found out that the application of three kinds of polymers (polyacrylic acid, polyvinyl alcohol, urea-formaldehyde resin) made soil water stable aggregate content increase by 17.27% averagely and density decrease by 11.18%, and soil water holding capacity increase by 2.8 times [2] compared with control. Huang Zhanbin et al [3] and Yuan Xuefeng et al [4] found that the addition of polymer into soil could enhance the bonding force between particles which are easy to disperse, and form larger aggregate structure, and especially the aggregate ratio of particles larger than 1 mm increased rapidly. Yang Peiling found that under the constant water head, after the application of water retaining agent of polymer, both the saturated hydraulic conductivity and infiltration rate decreased [5].

The purposes of this paper are as follows: (1) To introduce a sort of sodium polyacrylate with ultra-high molecular to apply to the improvement of water holding capacity of sandy soil; (2) To study the effect of sodium polyacrylate on saturated hydraulic conductivity and permeability property of sandy soil through experiment; (3) Tentatively to propose the suitable application amount of sodium polyacrylate into sandy soil.

## Materials and methods

### Experimental materials

Experimental soil was collected from the 30 cm layer of soil surface at the exposition garden of national water saving irrigation engineering technology research center at Yangling, and the soil texture is sandy soil (sand content is 80.04%, silt content is 14.31%, and clay content is 5.65%). The soil sample was naturally dried (mass soil water content is 0.45%) and then sieved with a sieve of 2 mm for reservation.

Sodium polyacrylate (SP for short) was produced and provided by Northwestern polytechnical university, which is spherical and translucent crystal particle, which is colorless and tasteless, and of uniform size (diameter is 0.2 mm or so) and whose molecular weight is 15 million-20 million.

### Experimental design and determination method

Sodium polyacrylate (SP) and dry soil should be well mixed by the weight ratio of 0, 0.08%, 0.2%, 0.5% and 1% respectively, and then five treatments of various concentrations can be obtained (C = 0, 0.08%, 0.2%, 0.5%, 1%, denoted by CK, SP_0.08_, SP_0.2_, SP_0.5_ and SP_1_ respectively). During the experimental process the soil bulk density should be 1.5 gcm^-3^.

The moisture retention curve of sandy soil was determined by use of centrifuge method. Soil treated with SP by different concentrations was fully saturated and then put in the high speed centrifuge to determine water content *θ* [6] under various water potential (centrifugal force) *S*.

Saturated hydraulic conductivity was determined by use of the constant water head method with double ring sampler. Soil treated with SP of various concentrations was put into ring sampler by the bulk density of 1.5 gcm^-3^, and then after full saturation the final stable water conduction rate Ks was determined under the water head (2 cm) controlled by flask Ma.

The infiltration experiment was carried out by use of one-dimensional soil column experiment. Soil treated with SP of various concentrations was put into the transparent glass tube by fixed bulk density layer by layer of 5 cm (height of glass tube is 70 cm, and inner diameter is 5.3 cm), and the soil height was 60 cm. Water was supplied by flask Ma and the constant water head was 2 cm. The water level scale and wetting front distance Z of flask Ma should be recorded every certain time. When the wetting front distance was 50 cm or so, the soil column was placed horizontally and then soil water content of different profile (every 2.5 cm) was determined by use of drying method.

## Results and analysis

### Effect on water-holding capacity of sandy soil

**Figure 1:**
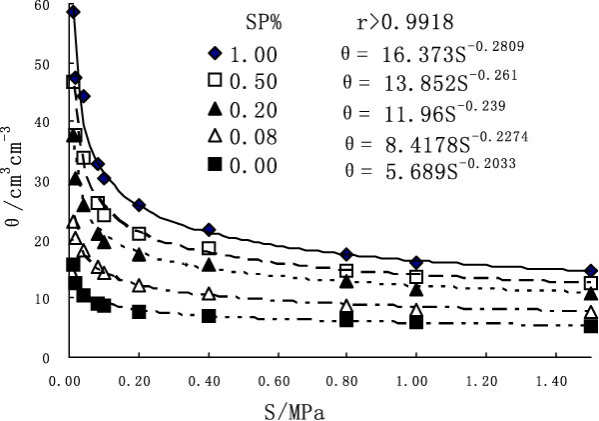
**Effects of treatments on water retention**.

The water-holding capacity graph of sandy soil under treatments of various concentrations of SP is shown as Figure 1. The graph shows that under the same water suction *S*, soil water retention increase under the influence of sodium polyacrylate compared with control, and the more the amount of sodium polyacrylate (SP%) is, *the bigger the* soil water retention *θ is*. Meanwhile it is also shown that under different treatments of SP water content *θ and water suction S both meet a* very significant(P = 0.01) power function relationship:

1

In the formula: *θ represents soil water content, and the unit is* cm^3^cm^-3^; *S represents water suction, and the unit is* Mpa; a and b are constant.

With the amount of SP increasing, the value of a increases. Through experiment Zhao Shiwei [7] found that the larger the value of a is, the stronger the soil water holding capacity is, which also demonstrates that the application of SP enhanced the soil water holding capacity, and the larger amount, the stronger capacity.

Under four treatments (SP_0.08_, SP_0.2_, SP_0.5_, SP_1_) the maximum capillary capacity*θ*_*0.01*_ (soil water retention, when *θ = 0.01*Mpa) increases by 45.7%, 138.6%, 195.7% and 271.8% respectively compared with CK; the wilting water level *θ*_*1.5*_ (soil water retention, when *θ = 0.01*MPa) increases by 5.9%, 107.7%, 137.8% and 177.9% separately compared with control. And that is the application of SP increases the upper limit of available soil water content and also increases the lower limit of unavailable water content. Here the *calculation formula* for the maximum water supply quantity *θ*_*a*_*has been* introduced *as follows:*2

In the formula: *θ*_*a*_*represents the maximum water supply quantity, and the unit is* cm^3^cm^-3^; *θ*_*0.01*_*andθ*_*1.5*_*represents* soil water contents under the water suctions of 0.01 Mpa and 1.5 Pma *respectively, and the unit is* cm^3^cm^-3^.

**Figure 2:**
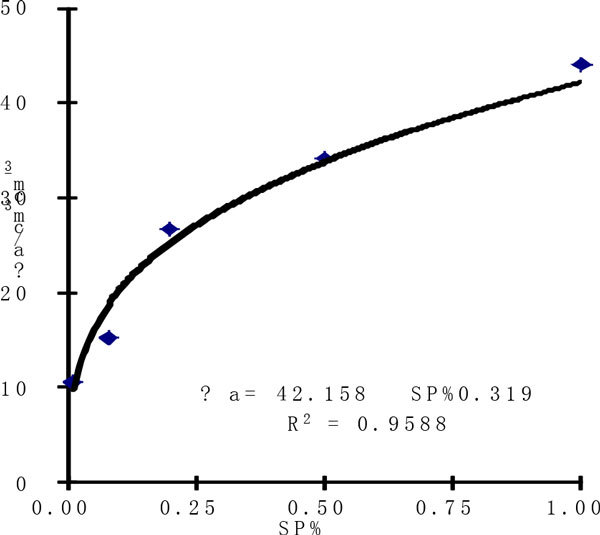
**Effects of water availability of SP of sand**.

The relationship between *θ*_*a*_*and the amount of* SP has been given as Figure 2, which shows that *θ*_*a*_*increases with the amount of* SP increasing, *and there exists a very significant power function relationship* (p = 0.01) between them. Under the four treatments (SP_0.08_, SP_0.2_, SP_0.5_, SP_1_) the maximum water supply quantity increases by 45.61%, 154.07%, 224.71%, 318.89% respectively compared with CK.

### Effect on saturated hydraulic conductivity

Sp significantly decreased the saturated hydraulic conductivity Ks of sandy soil and with the application amount increasing the decreased degree increased and there exists a very significant (p = 0.01) exponential relationship between Ks and the concentration of SP (*SP%*).3

In the formula: *Ks represents saturated hydraulic conductivity, and the unit is* cmh^-1^; *SP% represents the application amount of* SP; Under the treatments of SP_0.08_, SP_0.2_, SP_0.5_ and SP_1_, the Ks decreased by 42.53%, 55.45%, 87.55% and 96.5% respectively compared with CK. The cause of the decrease of Ks may be that the water absorption by SP and then the expansion decreased the paths for infiltration of water, and secondly the viscosity of SP itself become bigger, which increased the resistance against the migration of water downward in the soil, and the final result is presented as the decrease of Ks. That is the application of SP into sandy soil can decrease the infiltration capacity of water into deep layer under the saturation state, and the larger the application amount is, the larger the decreased degree is, which is helpful to store water within a certain arable layer.

### Effect on the migration of wetting front

Through the determination of the downward migration distance *Z of the* wetting front of unit time, it has been found that SP delayed the forward speed of wetting front, and that is after the application of SP the time needed by wetting front to reach a certain distance was prolonged. The relationship between the forward distance *Z* and the time t of wetting front has been given as follows:4

In the formula: *Z represents the forward distance of* wetting front, and the unit is cm; *t represents time*, and the unit is min; *a and b are constants*. To *make* integration of time *t in the* formula (4) and then the calculation formula for the infiltration rate of wetting front was obtained:5

Effect of treatments of SP of various amounts on infiltration distance of wetting front has been shown in Table 1, which shows that with the amount of SP increasing the value of a decreases accordingly, and the smaller the value of a is, the smaller the forward distance of wetting front is. *With the exception of* CK the value of *b exhibits the* same rule as the value of *a, and that is it decreases with the amount of* SP increasing. The formula (5) is the expression for the forward rate of wetting front.

**Table 1:** Effects of treatments on the advance of wetting front

Treatments	***Z = at*** ^***b***^	***R*** ^***2***^	***a ⋅ b***
CK	*Z = 7.30t* ^*0.6*^	0.9975	4.36
SP_0.08_	*Z = 5.27t* ^*0.65*^	0.9859	3.45
SP_0.2_	*Z = 4.81t* ^*0.64*^	0.9924	3.06
SP_0.5_	*Z = 4.27t* ^*0.5*^	0.9961	2.13
SP_1_	*Z = 1.75t* ^*0.39*^	0.9840	0.69

In the formula: the value of *a ⋅ b* basically determines the value of *dZ/dt* (the forward rate of wetting front). It has been shown in table 1 that the value of *a ⋅ b significantly decreases with the application amount of SP increasing, and that is to say the application of* SP significantly decreases the migration rate of wetting front, and the larger the *application* amount of SP is the smaller the migration rate of wetting front is, and also the time needed by wetting front to reach a certain distance. For instance, as for the treatments of CK, SP_0.08_, SP_0.2_, SP_0.5_ and SP_1_, under the irrigation condition of small amount of high drops on the surface, the time needed by wetting front to reach the distance of 20 cm are 5.4, 7.7, 9.4, 22.1 and 498.9 min respectively, which increases by 42.59%, 74.07%, 309.26% and 9138.89% respectively compared with CK.

### Effect on water content of soil profile

**Figure 3:**
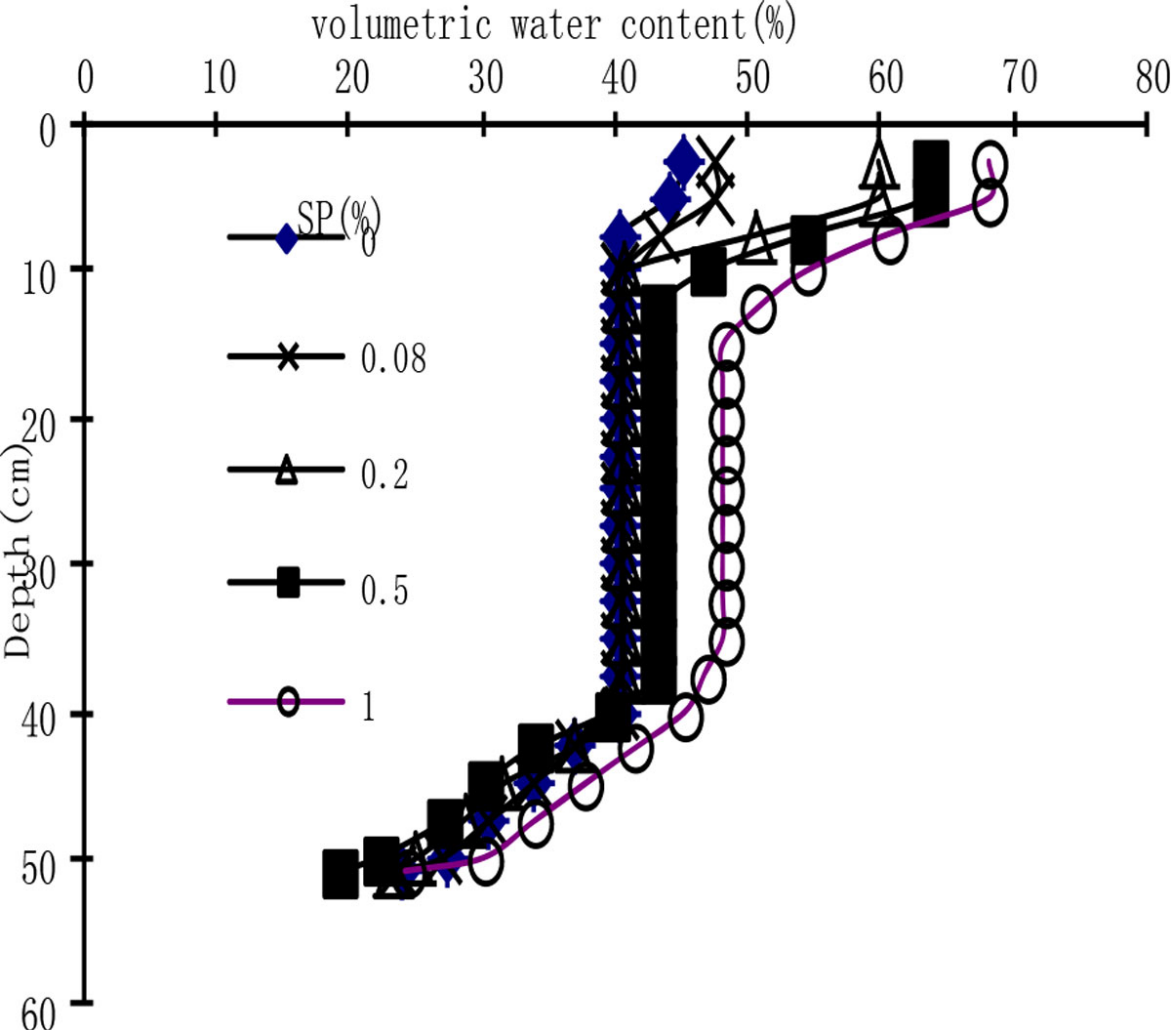
**Distribution of water content in the soil profile**.

When the wetting front reaches around the distance of 50 cm, the distribution of water content of soil profile is given as Figure 3. Figure 3 shows that the water distribution rule is consistent with the water distribution condition of homogeneous soil profile described by Huang Changyong [8]. As for the five treatments the water content of the surface soil of 0-5 cm is close to that of the position of 0 Mpa in Figure 1, and that is to say a saturated layer is formed; the position of 5-10 cm is transition region, where the water content changes more violently; 10-40 cm is extension layer, and within this layer the water content is almost a stable value; within 40-50 cm the water content decreases gradually, which can be seen as a wet layer and the wetting zone. The water content of the saturated layer and the transition region significantly increases with the application amount of SP increasing. Within the extension layer the difference of water content is small, and as for SP_1_ the water content increases in a relatively high degree compared with other treatments, while under the four treatments(CK, SP_0.08_, SP_0.2_ and SP_0.5_) the water content is similar; Within the wet layer the water content decreases with the soil depth increasing, and as for the various treatments the condition of water content at the wetting front is relatively complicated, and the order of water content at the wetting front under five treatments is SP_1_>CK>SP_0.08_>SP_0.2_>SP_0.5_.

### Effect on infiltration

The effect of SP on cumulative infiltration of sandy soil can be theoretically calculated through formula (6):67

In the formula: i *represents the cumulative infiltration within a certain time, and the unit is* mm; *D*_*i*_*represents the initial quantity of soil water deficit, and the unit is* cm^3^cm^-3^, where the water content of saturated layer, extension layer and wet layer of the surface soil is averaged into the water content of extension layer, and formula (7) is used to make the calculation, *and the unit is* cm^3^cm^-3^;*θ*_*T*_*represents the water content of the extension layer, and the unit is* cm^3^cm^-3^;*θ*_*i*_*represents the initial soil water content, and the unit is* cm^3^cm^-3^.

Formulas (4), (6) and (7) have been combined to obtain the theoretical formula (8) for calculating the cumulative infiltration:8

**Figure 4:**
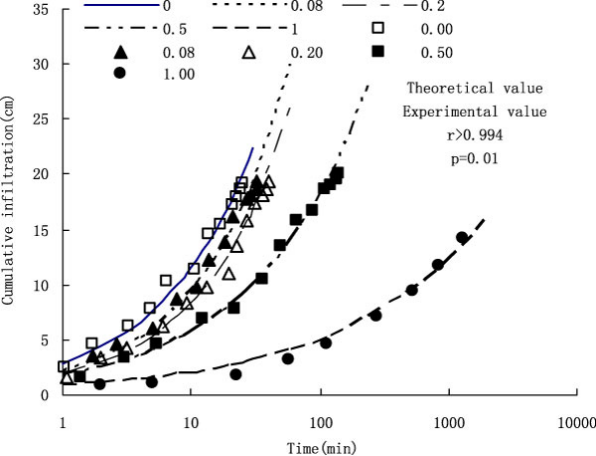
**Effects of treatments on cumulative infiltration**.

The actual cumulative infiltration was obtained through reading the flask Ma. The changing process of the measured value and theoretical value of cumulative infiltration with time under various treatments has been shown as Figure 4. Figure 4 indicates that the correlation between the measured value and theoretical value of cumulative infiltration is very significant (p = 0.01), and as long as any two parameter values among the values of *D*_*i*_*, I and Z are* known, the theoretical value of another parameter can be *derived* through the formula *i = D*_*i*_*× Z. As* the correlation between the theoretical value and measured value is especially high, the theoretical value can directly replace the measured value. Under the five treatments the increasing rates of cumulative infiltration all decreases with the prolongation of time, and that is to say during the initial stage of infiltration the increasing rate of cumulative infiltration is faster, and then the increasing rate slows down, which is consistent with the forward rule of wetting front. Meanwhile it can be seen that time needed by the same cumulative infiltration is prolonged with the application amount of SP increasing. For instance, as for the treatments of CK, SP_0.08_, SP_0.2_, SP_0.5_ and SP_1_ time needed by the infiltration amount of 10 cm are respectively 7.8, 10.5, 13.8, 32.0 and 637.5 min, and in terms of the treatments of SP_0.08_, SP_0.2_, SP_0.5_ and SP_1_ time needed increases by 33.7%, 75.9%, 308.5% and 8045.7% respectively compared with control.

The relationships of theoretical value and measured value of cumulative infiltration *i* with time *t both* meet the power function *i = at*^*b*^, and the infiltration rate *I* = *di/dt also* meets the power function relationship *I = mt*^*n*^, and the expressions for various treatments are shown in Table 2.

**Table 2:** Effects of treatments on the parameters infiltration rate

Treatments	***I = mt*** ^***n***^		r	
	**Theoretical value**	**Experimental value**	**m**	**n**
CK	*I = 1.733t* ^*-0.4*^	*I = 1.757t* ^*-0.418*^	0.997**	0.957*
SP_0.08_	*I = 1.371t* ^*-0.35*^	*I = 1.479t* ^*-0.413*^		
SP_0.2_	*I = 1.222t* ^*-0.36*^	*I = 1.198t* ^*-0.363*^		
SP_0.5_	*I = 0.905t* ^*-0.5*^	*I = 0.871t* ^*-0.449*^		
SP_1_	*I = 0.326t* ^*-0.61*^	*I = 0.24t* ^*-0.544*^		

** Correlation is significantly different at the 0.01 level; *Correlation is significantly different at the 0.05 level.

Table 2 shows that the coefficients m of theoretical value and measured value both decrease with the application amount of SP increasing, and the larger the value of m is, the larger the infiltration rate I is, and thus the infiltration rate decreases with the application amount of SP increasing. The value of n is negative, which shows that the value of I decreases with t increasing, and that is to say the infiltration rate decreases with the prolongation of infiltration time. Through the comparison of the correlation between the measured value and the theoretical value, it can be seen that the correlation coefficient of the main parameter m influencing the function value is 0.997, and the significant level is 0.01, and as for the parameter n the correlation coefficient is 0.957, and the significant level is 0.05. That is there is higher consistency between the measured value and the theoretical value of infiltration rate I.

## Conclusions and discussions

The following main conclusions have been drawn through the indoor simulation test for sodium polyacrylate: (1) The application of sodium polyacrylate can increase the water-holding capacity of sandy soil under different water potentials, including the maximum capillary water content and the wilting water level, and under the same water potential the larger the application amount of sodium polyacrylate is, the larger the water-holding capacity of sandy soil is, and as for the treatment of 0.08% the increasing amount of application amount is significantly lower than the three other treatments; (2) Sodium polyacrylate effectively enhances the maximum water storage and water supply capacity of sandy soil, and water supply capacity increases in power function with the application amount increasing, and as for the treatment of 0.08% the increasing amount of application amount is significantly lower than the three other treatments; (3) Sodium polyacrylate significantly decreases the saturated hydraulic conductivity of sandy soil, and the larger the application amount is, the larger the decreasing degree of saturated hydraulic conductivity is; (4) Under the infiltration condition of constant water head sodium polyacrylate decreases the forward rate of wetting front, and that is to say it decreases the migration velocity of water to the deep layer, and meanwhile the infiltration rate of sandy soil is decreased. The extremely low infiltration rate of the treatment of 1% completely hinders the infiltration of water into soil, and thus the application amount of 1% is too high, which is not suitable for popularization and application; (5) Under the condition of sufficient water supply for the surface soil sodium polyacrylate can increase the water content of saturated layer of the surface of sandy soil, but the effect for the extension layer is not significant, and thus the effect of applying sodium polyacrylate in various soil depths should be considered. (6) Based on the comprehensive consideration of the effect of sodium polyacrylate on the water storage capacity, the water supply capacity and the infiltration properties of sandy soil, the suitable application amount should be 0.2%-0.5%.

The application of sodium polyacrylate into the whole soil profile enhances the water storage capacity of the surface soil, and thus more water stays within the surface soil, and then the evaporation amount increases accordingly. Therefore it is recommended to move the application depth properly to the root zone under the soil surface. On the one hand, it is beneficial for the water infiltration from the surface soil to the root zone, and on the other hand it is helpful to decrease the water content of the surface soil and further to decrease the invalid evaporation. The further experimental study for the comprehensive effect of applying sodium polyacrylate into different soil depths will be needed.
